# Extracellular vesicle–cytokine communication in diabetic kidney disease: an integrated network linking renal resident cells and macrophages

**DOI:** 10.3389/fimmu.2026.1967300

**Published:** 2026-10-08

**Authors:** Zhixun Guo, Xinying Si, Zhichao Ruan, Jiangteng Liu, Nan Tong, Ying Tang, Yixin Ma, Dongchen Yang, Weijun Huang, Jinxi Zhao

**Affiliations:** 1 Dongzhimen Hospital, Beijing University of Chinese Medicine, Beijing, China; 2 Sunsimiao Hospital, Beijing University of Chinese Medicine, Tongchuan, Shaanxi, China; 3 Department of Endocrinology, The Affiliated Hospital of Jiangxi University of Chinese Medicine (Jiangxi Provincial Hospital of Traditional Chinese Medicine), Nanchang, China; 4 The First Affiliated Hospital of Zhejiang Chinese Medical University (Zhejiang Provincial Hospital of Chinese Medicine), Hangzhou, Zhejiang, China; 5 Key Laboratory of Chinese Internal Medicine of Ministry of Education and Beijing, Dongzhimen Hospital Affiliated to Beijing University of Chinese Medicine, Beijing, China

**Keywords:** cytokine networks, diabetic kidney disease, extracellular vesicles, immune cells, intercellular communication, intervention, resident kidney cells

## Abstract

Chronic inflammation contributes to the onset and progression of diabetic kidney disease (DKD), yet a model centered solely on soluble cytokines does not fully explain the cellular origin, spatially directed delivery, or sustained amplification of local signals. Extracellular vesicles (EVs) couple vesicular signaling with cytokine regulation through EV-associated cytokine or ligand signals and through RNA or protein cargo that reprograms cytokine production in recipient cells. This review examines how metabolic stress alters EV release and cargo composition, summarizes evidence for unidirectional and bidirectional communication between resident kidney cells and immune cells, and evaluates the EV specificity and evidential completeness of each donor–recipient axis. Evidence is strongest for communication between tubular epithelial cells and macrophages; findings involving podocytes, mesangial cells, and glomerular endothelial cells are largely unidirectional or inferred across studies. Under the *in vitro* polarization conditions used in the source studies, EVs from macrophages polarized toward a pro-inflammatory phenotype generally promote injury, whereas some EVs from macrophages polarized toward a reparative phenotype limit cell damage or improve autophagy. On this basis, we propose an EV-associated inflammatory communication network linked by shared inflammatory endpoints and inferred return signals. Within this network, we propose that injury-promoting and protective signals compete dynamically and thereby influence persistent inflammation and fibrosis-related disease progression. This hypothesis integrates existing evidence and offers a framework for understanding persistent inflammation in DKD and for testing therapies that target intercellular communication.

## 1 Introduction

Extracellular vesicles (EVs) are lipid-bilayer particles released by cells (1). Their cargo reflects the functional state of the cell of origin and may include proteins, nucleic acids, and lipids; EVs can alter recipient-cell function through surface ligand-receptor interactions, membrane fusion, or endocytosis (2–4). This regulated mode of signaling contributes to tissue homeostasis and disease and is particularly relevant to diabetic kidney disease (DKD), in which chronic inflammation is a defining feature. DKD is among the most common microvascular complications of diabetes and a leading cause of kidney failure worldwide (5, 6). Current therapies slow the loss of kidney function by improving hemodynamics, correcting metabolic disturbances, and limiting excessive receptor activation (7, 8). Nevertheless, many patients have persistent proteinuria, tubulointerstitial inflammation, and fibrosis and ultimately progress to kidney failure (9, 10). Additional mechanistic insight is therefore needed. Chronic low-grade inflammation is a major driver of DKD onset and progression and may persist even after metabolic control improves (10–14). Soluble cytokines such as TNF-α, IL-1β, IL-6, and CCL2 recruit immune cells and amplify inflammation (15–18). Yet a soluble-mediator model alone cannot readily identify the cells producing a given inflammatory signal or explain how that signal remains stable in the extracellular milieu and reaches selected recipient cells. Nor can it readily account for the self-perpetuation of local inflammation (19). EV cargo reflects the state of its source cell, the vesicular membrane protects some cargo, and surface molecules can confer partial recipient-cell selectivity. Together, these properties provide a basis for stable local signal transfer.

Both resident kidney cells and immune cells release EVs. Inflammation-related EV signals can be grouped into three broad classes: vesicles that directly carry cytokines or chemokines, signaling mediated by ligand-receptor interactions at the vesicle surface, and RNA or protein cargo that induces a secondary cytokine response in recipient cells (20–24). These mechanisms provide a signaling route distinct from soluble diffusion and may account for the stability and targeting that soluble-mediator models leave unexplained. However, most studies examine a single donor-recipient pair. Few validate both a forward and a return signal, and most proposed axes do not yet qualify as closed feedback processes. Previous reviews have largely organized the field by EV source, miRNA cargo, or biomarker application (25–30), with less emphasis on integrating bidirectional EV communication between resident kidney cells and immune cells as a continuous pathogenic process (31). Here, we use EV–cytokine communication as the organizing framework, encompassing direct EV-associated cytokine or chemokine signals, membrane-associated signaling, and EV cargo that reprograms cytokine production in recipient cells. We describe how metabolic stress reshapes EV release and cargo in DKD, review validated and putative bidirectional communication between resident kidney cells and immune cells, and distinguish direct evidence from cross-study inference when assembling individual axes into a testable pathogenic network. In this review, EV–cytokine communication is defined as a network in which kidney resident cells and immune cells are the nodes and EV-associated signals form the donor–recipient connections. Several of these connections are supported by existing studies. Whether they influence one another through return signals and changes in cell state remains a hypothesis. We therefore grade the evidence for each connection and distinguish unidirectional effects, bidirectional communication validated within a single experimental system, and relationships inferred across separate studies. This framework also provides a route to distinguish parallel responses to a shared metabolic stimulus from coordinated communication with cross-axis dependencies.

## 2 Extracellular vesicles as mediators of intercellular signaling

As intercellular signal carriers, EVs are distinguished by selective cargo loading, protection of cargo within a membrane-bound compartment, and a degree of selectivity for recipient cells. Source cells actively sort particular proteins, lipids, and RNAs into vesicles, producing a cargo profile that differs from the overall composition of the parent cell. Several cytokines are secreted in this selectively packaged form, and for the best-studied example trypsin protection and detergent lysis established that the cytokines are enclosed within the vesicles (19, 23). The lipid bilayer shields cargo from extracellular proteases and nucleases, stabilizing cytokine signals that would otherwise be rapidly diluted or degraded. On reaching a recipient cell, an EV may initiate signaling through a surface ligand-receptor interaction or deliver its cargo by endocytosis or membrane fusion (2, 3). Interactions between EV surface molecules and components of the recipient-cell membrane can also confer partial cellular selectivity (20–22). EVs can therefore shape the composition, stability, and spatial range of a signal in ways that differ from freely soluble cytokines (24). In accordance with the Minimal Information for Studies of Extracellular Vesicles (MISEV) guidelines, we use extracellular vesicle as the default term. We retain subtype labels such as exosome or microvesicle only when the original study provides evidence for the corresponding biogenesis pathway or when the label is needed to report the authors’ terminology faithfully. Macrophage states are reported as pro-inflammatory or reparative rather than as M1 or M2. Our assessment of each communication axis considers EV characterization, EV-depleted or other appropriate controls, protease- or nuclease-protection experiments, functional perturbation of the cargo or receptor, and, when available, *in vivo* source tracing. The per-study appraisal of these criteria is provided in **Supplementary Table 1**. Findings that lack key causal evidence are described as EV-associated effects rather than confirmed vesicular cargo transfer, which helps limit confounding by soluble cytokines, protein aggregates, and other co-isolated material (1, 32).

Under physiological conditions, EVs contribute to kidney development, maintenance of the glomerular filtration barrier, and fluid and electrolyte homeostasis (33). The resulting intercellular communication is cell-type- and context-dependent rather than indiscriminate disposal of cellular material (34). In collecting-duct models, for example, EVs from vasopressin-stimulated donor cells transfer functionally active aquaporin-2 (AQP2) to recipient cells, and desmopressin increases EV uptake by collecting-duct cells (35). These findings establish the biological feasibility of regulated EV communication within defined nephron segments. In DKD, high glucose, albumin overload, and lipotoxicity can further alter EV release and cargo, shifting some communication axes toward pro-inflammatory and injury-promoting effects. Such changes form a shared upstream context for pathological EV remodeling, whereas the physiological studies provide the conceptual baseline against which that remodeling can be understood.

## 3 Metabolic stress reshapes EV signaling and the cytokine microenvironment

Metabolic stress alters both the amount and composition of EVs released by resident kidney cells and can thereby modify their signaling to immune cells. High glucose increases podocyte EV release through the NOX4/ROS pathway (36). EVs from high-glucose-treated glomerular endothelial cells induce an epithelial-to-mesenchymal-transition-like phenotype and functional injury in podocytes (37), while high glucose also changes mesangial-cell EV composition and enriches cargo related to the renin-angiotensin-aldosterone system, including angiotensinogen (38). Under albumin overload, tubular epithelial cell (TEC)-derived EVs stabilize hypoxia-inducible factor 1α (HIF-1α) and enhance glycolysis in macrophages. Candidate miRNAs and lncRNAs associated with HIF-1α regulation are enriched in these vesicles, but their individual contributions remain unresolved because cargo-specific perturbation and rescue experiments have not been performed (39). Under lipotoxic conditions, leucine-rich alpha-2-glycoprotein 1 (LRG1) is enriched in TEC-derived EVs and is associated with transforming growth factor-β receptor 1 (TGFβR1)-dependent macrophage activation (40). Thus, metabolic injury can redirect EV-mediated communication from homeostatic regulation toward pro-inflammatory and injury-promoting signaling (**Figure 1**).

**Figure 1:**
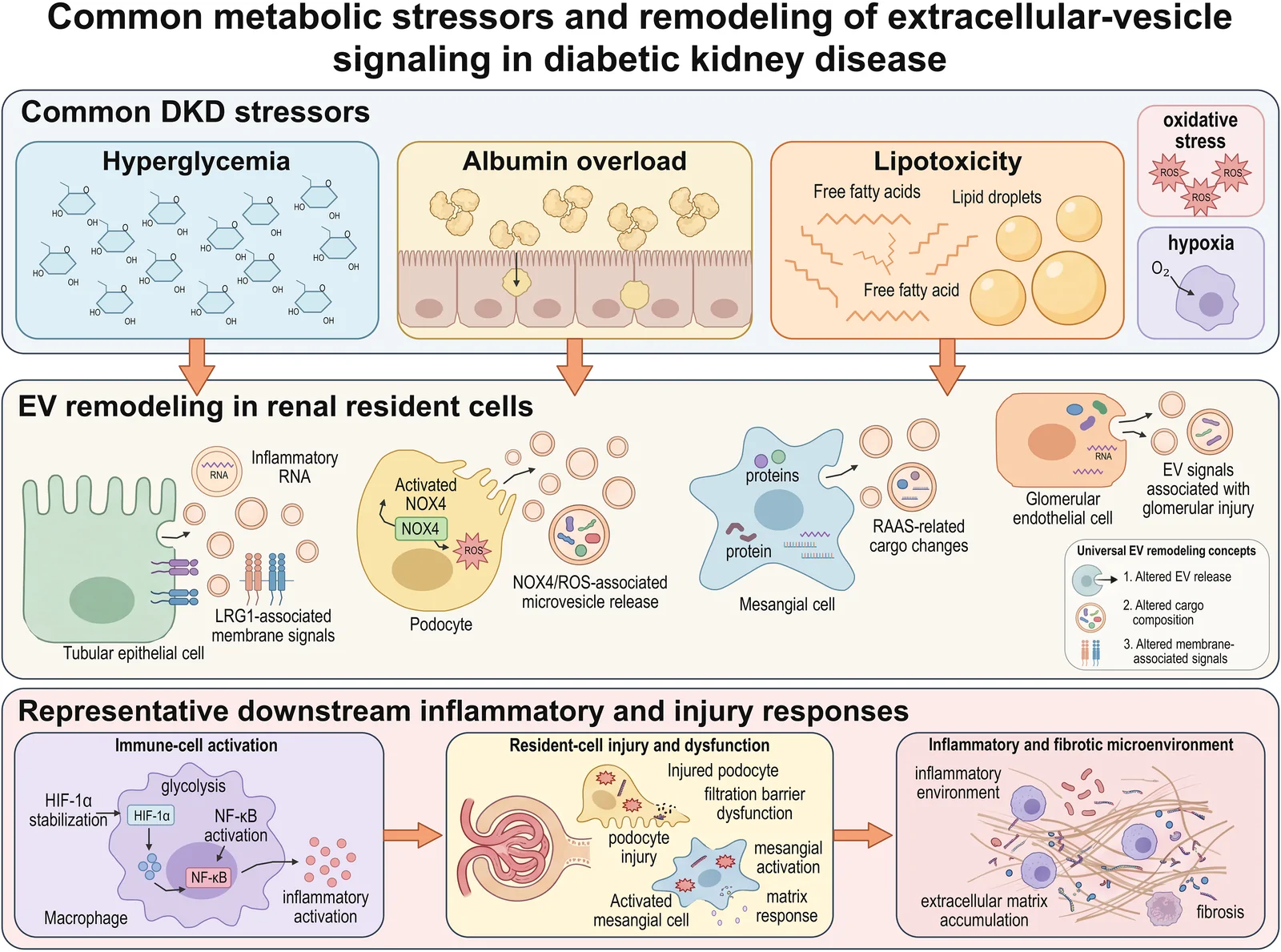
Common metabolic stressors and remodeling of extracellular-vesicle signaling in diabetic kidney disease. Hyperglycemia, albumin overload, lipotoxicity, oxidative stress, and hypoxia alter EV release, cargo composition, and membrane-associated signals in tubular epithelial cells, podocytes, mesangial cells, and glomerular endothelial cells. Representative downstream responses include macrophage glycolytic and inflammatory activation, injury to resident kidney cells and the glomerular filtration barrier, extracellular-matrix accumulation, and fibrosis. EV, extracellular vesicle; GEC, glomerular endothelial cell; HIF-1α, hypoxia-inducible factor 1α; LRG1, leucine-rich alpha-2-glycoprotein 1; NOX4, NADPH oxidase 4; RAAS, renin–angiotensin–aldosterone system; ROS, reactive oxygen species.

Metabolic stress changes not only EV abundance but also EV cargo, which can engage inflammatory pathways in recipient cells. The mRNA and protein profiles of urinary EVs vary with DKD phenotype or stage, supporting their potential as readouts of disease-related stress and as candidates for disease stratification and biomarker studies (41, 42). Macrophages are prominent immune effector cells in the inflammatory microenvironment of the DKD kidney, and their subset composition and activation states change as disease progresses (43). RNA and protein cargo carried by EVs can activate NF-κB and increase expression of inflammatory mediators such as IL-6 in recipient cells, thereby intensifying local inflammation (44, 45). This mechanism recurs across several axes linking resident kidney cells to immune cells. Evidence from other diseases and from nondiabetic kidney injury provides a mechanistic reference for bidirectional EV communication. In prostate cancer, tumor-derived EVs transfer miR-181a-5p to drive M2 macrophage polarization, while taurine released by M2 macrophages acts back on tumor cells to inhibit ferroptosis, forming an experimentally validated positive feedback loop (46). In nondiabetic kidney injury such as unilateral ureteral obstruction, EV-mediated communication between TECs and macrophages can amplify tubulointerstitial inflammation and fibrosis in both directions, and inhibition of EV release interrupts this process (47). These models are relevant because they show that EVs from one cell population can alter an immune-cell phenotype and that the immune cell can then signal back through EVs or soluble mediators. They support the biological feasibility of bidirectional EV communication and can inform the design of DKD-specific studies. They remain mechanistic analogies, however: differences in disease context and cellular microenvironment require the corresponding communication relationships and feedback loops to be tested directly in DKD.

## 4 EV-mediated resident-cell–macrophage communication axes, feedback loops, and cytokine networks

EV-mediated intercellular communication may sustain and amplify local inflammation in DKD, but most studies address isolated unidirectional pathways and do not systematically test whether the signals form feedback loops (25, 31). Given the basic sequence in which an EV transfers a signal and changes recipient-cell function (3, 22), three elements are required to evaluate a feedback loop. First, EVs from a defined donor cell must alter the phenotype of a recipient cell. Second, the altered recipient cell must act back on the original donor through EVs or another defined return signal. Third, interrupting both directions in the same disease model should attenuate the corresponding pathology. EV-associated return communication can therefore be graded as unidirectional EV communication, a bidirectional communication chain, or a candidate feedback loop. Unidirectional communication requires a defined donor EV to affect a specified recipient cell. A bidirectional chain requires both the forward and return directions to be validated within the same experimental framework. Only when the return signal further alters the initial donor or its subsequent EV output, allowing the sequence to recur, should the interaction be considered a candidate feedback loop. Cell-type-specific interruption of both directions *in vivo* would provide stronger causal evidence. By these criteria, the LRG1/tumor necrosis factor-related apoptosis-inducing ligand (TRAIL) axis is the most completely supported bidirectional pathogenic communication chain validated within a single experimental system in DKD (40). Most other proposed relationships rely on unidirectional evidence or integration across separate studies (**Table 1**; **Figure 2**).

**Table 1:** EV-mediated communication axes between resident kidney cells and immune cells in DKD.

Communication axis	Representative EV cargo or signal	Principal effect	Evidence source	Evidence appraisal	Inferential status	EV attribution
TEC → macrophage	miR-19b-3p, miR-199a-5p, miR-23a, Dll4, CCL2 mRNA, LRG1, and candidate HIF-1α-related RNAs	Stabilizes HIF-1α, enhances glycolysis, and activates NF-κB or Notch signaling and pro-inflammatory macrophage responses	DKD models and related tubular injury models	Most extensive directional evidence. The LRG1 arm is receptor-dependent, and its return arm was validated in the same experimental system; individual candidate RNAs remain unresolved	Same-study bidirectional (LRG1/TRAIL); other cargoes unidirectional	(39) (A1) (49), (A1) (40), (forward arm A1; return arm A2) (48), (A2) (50), (A2) (51), (A2) (53), (A2)
Kidney-tissue EV →macrophage	miR-34a	Promotes a pro-inflammatory macrophage state and is associated with a tubular fibrotic phenotype	DKD kidney-tissue EVs and cell experiments	Supports an EV-associated effect, but the donor cell was not lineage-resolved; not assigned to a defined TEC-to-macrophage axis	Unidirectional only	(52) (A2)
Macrophage → TEC	TRAIL, miR-7002-5p, and differentially expressed lncRNAs	Promotes TEC apoptosis, impaired autophagy, and phenotypic transition	DKD animal and cell models	TRAIL is the return arm of the LRG1/TRAIL bidirectional pathogenic chain validated in one experimental system; the other cargoes are independent return signals	Same-study bidirectional (LRG1/TRAIL); other cargoes unidirectional	(54) (A1) (40), (A2) (55), (A2)
Podocyte →macrophage	APOC1	Activates NF-κB and promotes pro-inflammatory macrophage activation	High-glucose human podocyte cell line, THP-1-derived macrophage model, and database analyses	Unidirectional *in vitro* evidence; lacks mechanistic animal validation and cell-of-origin-specific *in vivo* tracing	Unidirectional within each study; the pair is cross-study inferred	(57) (A2)
Macrophage →podocyte	miR-21-5p/miR-21a-5p,lncRNA AFAP1-AS1,and miR-25-3p	Promotes NLRP3-related pyroptosis or caspase-3-related apoptosis, or suppresses pyroptosis and improves autophagy	DKD-related cell and animal models	Direction of effect varies with the macrophage state defined in each study; cargoes were not compared directly in one model and do not establish a closed loop	Unidirectional within each study; the pair is cross-study inferred	(62) (A1) (58), (A2) (60), (A2) (61), (A2)
Mesangial cell →macrophage	Active cargo not identified	Activates NF-κB and enhances pro-inflammatory responses	High-glucose cell model and pharmacological intervention in animals	Supports directional EV communication; the key cargo and its specific causal contribution remain undefined	Unidirectional within each study; the pair is cross-study inferred	(63) (A2)
THP-1 monocyte →mesangial cell	Microvesicle-associated IL-6, IL-8, and MCP-1	Enhances HIF-1α/VEGF-related responses in mesangial cells	*In vitro* high-glucose THP-1 monocyte and human mesangial-cell model	Unidirectional effect associated with monocyte microvesicles; limited exclusion of co-isolates and cargo-topology testing lowers EV specificity	Unidirectional only	(64) (A3)
Macrophage →mesangial cell	TGF-β1 mRNA; NLRP3- and autophagy-related signals	Activates Smad3 and extracellular matrix deposition, or activates NLRP3 and impairs autophagy	DKD cell and animal models	Donor TGF-β1 knockdown in ref 65 supports its contribution to the EV-associated effect; NLRP3 is a recipient-cell response module	Unidirectional within each study; the pair is cross-study inferred	(65) (A2) (66), (A2)
Circulating or kidney-tissue EV signal →mesangial cell	miR-3147	Affects mesangial-cell proliferation and apoptosis under high glucose through PRKAR2B	Plasma EVs from patients with DKD, kidney-tissue localization, and functional experiments in mesangial cells	Supports disease association and an intracellular mesangial-cell mechanism, but does not establish mesangial-cell EV release, uptake, or autocrine transfer	Unidirectional only	(67) (A2)
Macrophage in a pro-inflammatory state →GEC	METTL14	Aggravates endothelial apoptosis, inflammation, and oxidative stress through PAQR3-related m6A regulation	*In vitro* high-glucose macrophage-GEC model	Unidirectional *in vitro* evidence	Unidirectional only	(68) (A1)
GEC → mesangial cell	TGF-β1 mRNA	Activates Smad3 and promotes mesangial-cell proliferation and extracellular matrix deposition	High-glucose GEC-mesangial-cell model with EV-directed intervention	Defined donor and direction in a unidirectional profibrotic pathway; not connected to the macrophage axis in the same system	Unidirectional only	(69) (A2)
Glomerular epithelial → mesangial cell	circTAOK1	Promotes mesangial-cell proliferation, fibrosis, and phenotypic transition through the miR-520h/SMAD3 axis	High-glucose cell model	Supports a unidirectional effect	Unidirectional only	(70) (A2)
Platelet microparticle → GEC	CXCL7	Promotes early glomerular endothelial injury	Clinical and experimental models of early DKD	Unidirectional injurious signal of circulating origin.	Unidirectional only	(71) (A2)
CD4+ T cell → TEC	Small-EV-associated signal and regulation of mitochondrial dynamics	Induces mitochondrial dysfunction and apoptosis in TECs	DKD cell and animal models	Source-defined unidirectional adaptive-immune communication; no evidence for a return arm	Unidirectional only	(72) (A2)

Inferential status: Same-study bidirectional, both directions tested in one experimental system; unidirectional only, a single direction with no counterpart row; or cross-study inferred, the two directions supported in separate studies but never connected. EV attribution: A1, EV-dependent effect supported by characterization, a valid vesicle-depleted control, and demonstrated uptake or transfer; A2, some of these; A3, not supported. These grades do not rank the completeness of the relationships, and the arrow styles in **Figure 2** follow the inferential-status levels. Per-study appraisal is in **Supplementary Table 1**.

**Figure 2:**
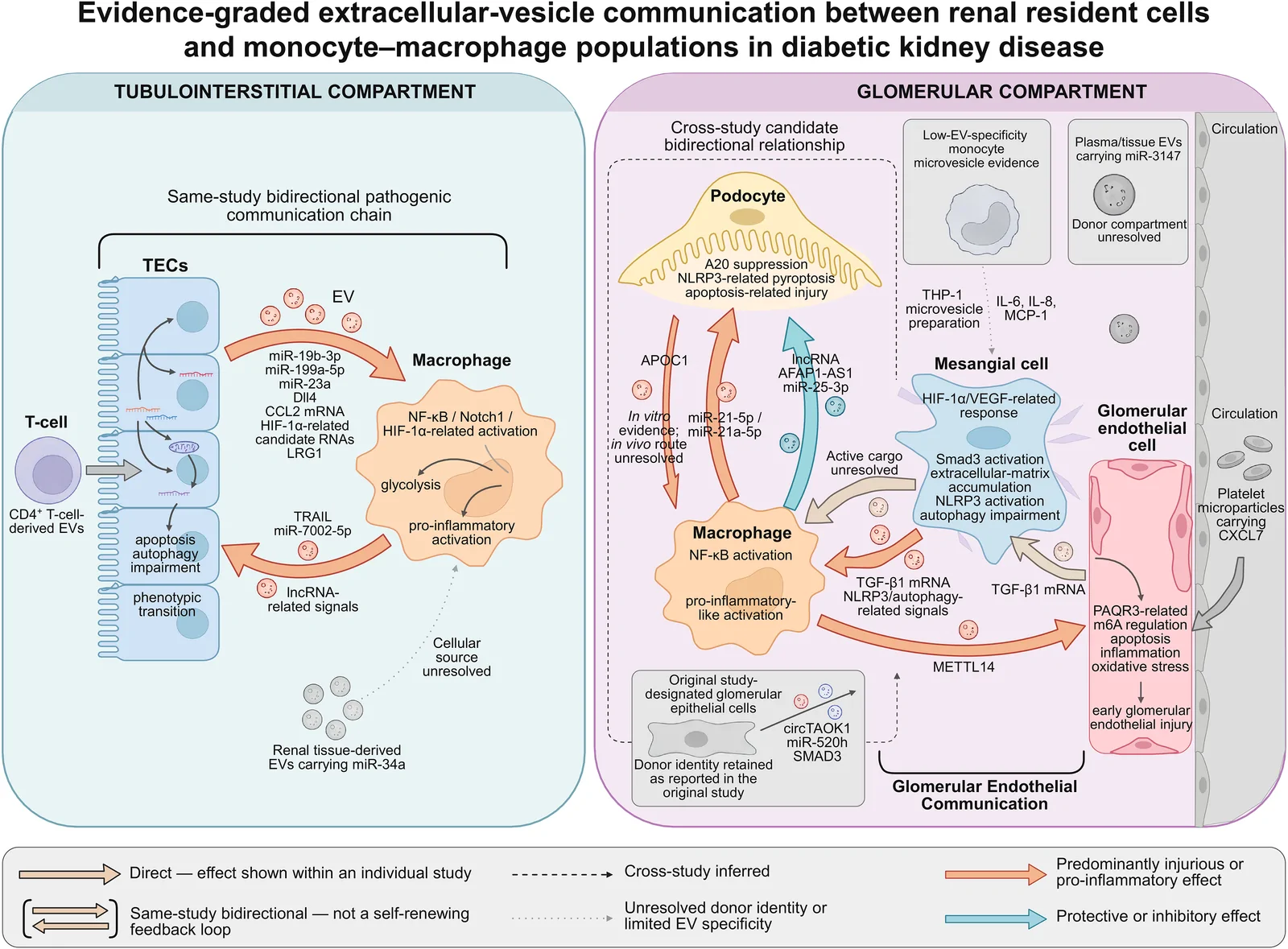
Qualitative evidence appraisal of extracellular-vesicle-associated communication between renal resident cells and monocyte–macrophage populations in diabetic kidney disease. Solid arrows denote a Direct inferential status: the effect was shown within a single study. Paired arrows joined by a bracket denote the LRG1/TRAIL chain, graded Same-study bidirectional in **Table 1**: both directions were tested in one experimental system, and the configuration is not interpreted as a self-sustaining feedback loop. Dashed arrows denote a Cross-study inferred status: the connection is inferred across separate studies. Gray symbols indicate indirect evidence, unresolved donor-cell identity, or limited EV specificity. Red or orange arrows denote predominantly injurious or pro-inflammatory effects, whereas blue arrows denote protective or inhibitory effects. APOC1, apolipoprotein C1; CCL2, C-C motif chemokine ligand 2; DKD, diabetic kidney disease; EV, extracellular vesicle; GEC, glomerular endothelial cell; LRG1, leucine-rich alpha-2-glycoprotein 1; TEC, tubular epithelial cell; TGF-β1, transforming growth factor-β1; TRAIL, TNF-related apoptosis-inducing ligand.

### 4.1 Communication between tubular epithelial cells and macrophages

Communication between TECs and macrophages is supported by the most extensive evidence. Within this literature, the LRG1/TRAIL axis is the best-supported bidirectional pathogenic chain because both the forward and return arms were tested in the same experimental system. During lipotoxic injury, TECs release LRG1-enriched EVs that induce macrophage expression of TNF-α, IL-1β, and C-X-C motif chemokine ligand 12 (CXCL12) through a TGFβR1-dependent mechanism and simultaneously increase macrophage EV release. Activated macrophages then release TRAIL-enriched EVs, and this is accompanied by upregulation of death receptor 5 (DR5) in injured TECs, although receptor blockade was not performed (40). Inhibition of EV uptake with cytochalasin D did not substantially reduce macrophage activation or TEC apoptosis, indicating that membrane-level interactions between EVs and recipient cells may predominate. Recombinant LRG1 likewise induced inflammatory cytokine expression and EV release in macrophages, whereas recombinant TRAIL combined with lipotoxic stress increased TEC apoptosis, supporting the biological activity of both molecules. Immunogold electron microscopy localized both ligands to the vesicles, and the forward arm was confirmed by TGFβR1 knockdown, identifying LRG1/TGFβR1 as an EV-associated, receptor-dependent signal. Lipotoxicity acts upstream of this chain, showing that metabolic stress can both remodel EV cargo and initiate pathogenic communication between TECs and macrophages.

Beyond the LRG1/TRAIL chain, several studies have described pro-inflammatory signals transmitted from TECs to macrophages by EVs. The implicated cargoes act through different mechanisms. Three TEC-derived EV cargoes relieve inhibition of NF-κB signaling and promote a pro-inflammatory macrophage state through distinct targets: miR-19b-3p suppresses SOCS1, miR-199a-5p suppresses Klotho, and miR-23a targets A20 (48–50), Delta-like ligand 4 (Dll4) acts mainly through Notch signaling (51). Both routes increase pro-inflammatory activation and mediator expression. Kidney-tissue-derived EVs containing miR-34a also promote polarization toward a pro-inflammatory macrophage state, although the specific donor cell has not been resolved (52). Two additional mechanisms are distinct: TEC-derived EVs transfer CCL2 mRNA and increase CCL2 expression in macrophages (53), while another TEC-derived EV signal stabilizes HIF-1α and enhances glycolysis to support pro-inflammatory activation (39). In the return direction, macrophage EVs released under experimentally pro-inflammatory or high-glucose conditions injure TECs through mechanisms other than TRAIL-mediated apoptosis. These include miR-7002-5p-mediated inhibition of TEC autophagy (54) and lncRNA-mediated induction of epithelial-to-mesenchymal transition (55). Collectively, macrophage-derived EVs aggravate TEC injury through apoptosis, impaired autophagy, and phenotypic transition. Together with pro-inflammatory signaling from TECs to macrophages, these return signals can contribute to the persistence and amplification of tubular inflammation and injury. TECs also propagate phenotypic transition within the tubular cell population through autocrine EVs (56), adding an intratubular component to the network (**Figure 2**). Of these axes, only LRG1/TRAIL has its forward and return actions connected within a single experimental system. This is the most completely supported communication relationship in this review and serves as the reference for the following sections.

### 4.2 Communication between podocytes and macrophages

Podocytes receive both injury-promoting and protective EV signals from macrophages in different activation states. Because these signals converge on related inflammatory and autophagic nodes but regulate them in opposite directions, their balance may influence podocyte fate. Evidence for the forward direction is based primarily on apolipoprotein C1 (APOC1) carried by podocyte-derived EVs, which activates macrophage NF-κB signaling and promotes pro-inflammatory macrophage activation (57). In the macrophage-to-podocyte direction, EVs from pro-inflammatory macrophages mainly promote podocyte injury through pyroptosis. Their miR-21-5p cargo enters podocytes, suppresses A20, augments NLRP3 inflammasome-related signaling, and increases podocyte pyroptosis and release of IL-1β and IL-18 (58, 59). Treatment with Yi-Shen-Hua-Shi granules reduced markers of pro-inflammatory macrophage activation, miR-21a-5p in macrophage EVs, and podocyte injury, providing intervention-based support for an association among macrophage state, EV cargo, and podocyte damage (60). By contrast, EVs from reparative macrophages carry cargoes that regulate the same molecular system in the opposite direction. lncRNA AFAP1-AS1 recruits EZH2 to silence NLRP3 transcription and suppress podocyte pyroptosis (61), while miR-25-3p restores podocyte autophagy by downregulating DUSP1 (62). These studies link injury-promoting and protective macrophage-derived EV signals to NLRP3-related pyroptosis and autophagy, with opposing effects at selected molecular nodes. Podocyte responses are likely to depend on macrophage activation state, EV cargo dose, exposure timing, and the state of the recipient cell. Macrophage activation can therefore shape both the composition and direction of the return EV signal. The evidence is weaker than for the TEC-macrophage axis: forward signaling (57) and return communication (58) were demonstrated in different experimental systems and have not been connected in a single model. Podocytes and kidney macrophages also occupy different anatomical compartments. The *in vivo* route of EV transfer between them remains uncertain, and no source-specific tracing has yet shown podocyte-derived EVs crossing the glomerular filtration barrier to reach macrophages or monocytes. The evidence in this section holds within each direction, but the two directions come from different studies and have not been connected within a single experimental system. The bidirectional relationship between podocytes and macrophages is therefore a connection inferred across studies rather than a validated communication chain.

### 4.3 Communication between mesangial cells and macrophages

In the forward direction, EVs from high-glucose-treated mesangial cells activate macrophage NF-κB signaling, but the active cargo has not been identified, which limits the strength of the evidence (63). A chemical screen targeting EV communication found that alvespimycin blocked EV transfer between mesangial cells and macrophages and improved the DKD phenotype, providing pharmacological support for this route (63). In the opposite direction, microvesicle preparations from high-glucose-stimulated THP-1 monocytes were reported to contain IL-6, IL-8, and MCP-1 and to enhance HIF-1α/vascular endothelial growth factor (VEGF) signaling in mesangial cells (64). Under the criteria applied here, this axis remains at the level of an EV-associated effect rather than a defined EV-mediated transfer.

Studies of macrophage-derived EVs have instead focused on pro-inflammatory and profibrotic effects. EVs from high-glucose-stimulated macrophages deliver TGF-β1 mRNA, activate Smad3 in mesangial cells, and increase extracellular matrix deposition (65). Other macrophage-derived EVs activate the NLRP3 inflammasome and impair autophagy (66). In addition, miR-3147 is increased in plasma EVs from patients with DKD and shows greater localization to the mesangial region in kidney tissue; *in vitro*, miR-3147 affects mesangial-cell proliferation and apoptosis under high-glucose conditions by targeting protein kinase cAMP-dependent type II regulatory subunit beta (PRKAR2B) (67). Existing studies therefore provide directional evidence in both directions, but the two arms have not been connected within the same experimental system and therefore do not establish a bidirectional chain or a feedback loop. As with the podocyte axis, the two directions here come from different experimental systems and constitute a connection inferred across studies. The available evidence supports two independent unidirectional relationships but is not sufficient to combine them into a single chain. The next step would be to test, within a single experimental system, whether the change in macrophage state induced by mesangial cell EVs in turn alters the EV cargo and functional output that these macrophages return to mesangial cells. Only after the forward stimulus, the receptor response, and the return action are connected in temporal sequence can the two directions constitute interdependent bidirectional communication (**Figure 2**).

### 4.4 EV communication involving glomerular endothelial cells and its network connections

Current studies involving glomerular endothelial cells (GECs) primarily describe unidirectional communication from macrophages to GECs and from GECs to mesangial cells. In the macrophage-to-GEC direction, EVs from macrophages in pro-inflammatory states carry METTL14 and regulate PAQR3 through N6-methyladenosine (m6A) modification, thereby increasing GEC apoptosis, inflammation, and oxidative stress under high-glucose conditions (68). Macrophage EV injury is therefore not confined to tubules and mesangium but extends to the endothelial side of the glomerular filtration barrier. In the GEC-to-mesangial-cell direction, EVs from high-glucose-treated GECs deliver TGF-β1 mRNA, activate Smad3, and promote mesangial-cell proliferation and extracellular matrix deposition (69). A separate study reported that EVs from what the original authors termed “glomerular epithelial cells” contained circTAOK1, which increased SMAD3 by sponging miR-520h and thereby promoted mesangial-cell proliferation, fibrosis, and phenotypic transition (70). This finding is presented here as independent evidence from a different donor source. Integrated with the directional evidence between mesangial cells and macrophages, these findings suggest that GEC signals connect functionally with other inflammatory axes through mesangial cells, although this cross-axis connection remains an inference drawn across separate studies. Circulating platelet microparticles carrying CXCL7 also contribute to early GEC injury (71), showing that some EV-associated signals reaching GECs originate outside the kidney. GECs may therefore be better viewed as a glomerular communication node that receives injurious signals from macrophages or the circulation and transmits profibrotic signals to mesangial cells (69). In this role, GECs function as both recipients and donors within EV communication. This section brings glomerular endothelial cells into the account, and each of the relationships described here is unidirectional.

### 4.5 An integrated EV–cytokine network model in DKD

We propose that resident kidney cells influence one another through macrophages and shared inflammatory and fibrotic mediators, forming a communication network in which EV signals and soluble cytokines act together. After entering recipient cells, EV cargo can induce inflammatory mediator expression. EVs amplify local inflammation through two distinct mechanisms. Ligands displayed at the vesicle surface engage receptors on the recipient cell, as established for LRG1 and TRAIL in the tubular–macrophage axis (40), whereas RNA cargo delivered into recipient cells reprograms their cytokine production, as shown for TGF-β1 mRNA and for several microRNAs and long non-coding RNAs (48, 49, 57). Soluble mediators, including TNF-α, IL-1β, IL-6, CCL2, CXCL12, and TGF-β1, diffuse between cells. EV signaling and the local cytokine milieu can influence one another: EVs reshape the microenvironment by altering inflammatory mediator expression in recipient cells, whereas changes in inflammatory and metabolic state can modify EV release, cargo sorting, and uptake (23, 24, 36, 37, 39, 40). This reciprocal relationship provides a basis for integrating otherwise separate communication axes. High glucose, albumin overload, and lipotoxicity can simultaneously alter EV release and cargo in several kidney cell types and thereby initiate or strengthen multiple inflammatory and injury-promoting pathways. These stressors can thus be considered major upstream drivers of the network. Functional convergence at a limited set of inflammatory and fibrotic nodes provides further support for network-level connections. Forward signals from TECs, podocytes, and mesangial cells can all converge on NF-κB-related pro-inflammatory responses in macrophages (48–50, 57, 63). Macrophage centrality is the organizing hypothesis of this review rather than an experimentally established property; it also reflects the fact that most available studies used macrophages as the donor or recipient population. TGF-β1 is both macrophage EV cargo and a shared driver of fibrosis in several resident kidney cell types (65, 69), while IL-1β- and NLRP3-related responses occur in podocyte- and mesangial-cell-associated axes (58, 66). Thus, distinct communication pathways converge within recipient cells on shared NF-κB, NLRP3, and TGF-β/Smad effector modules. This convergence creates functional coupling at the level of downstream effects and offers a framework for considering the aggregate pathological influence of an EV-associated cytokine network in DKD.

The proposed network also extends beyond its central chains. TEC-derived EVs promote pro-inflammatory macrophage activation (48), and EVs released by activated macrophages can then injure podocytes (58). Although derived from different experimental systems, these findings suggest a functionally linked three-cell path involving TECs, macrophages, and podocytes that could connect tubulointerstitial inflammation with glomerular injury. Its temporal sequence and *in vivo* transfer route in DKD remain untested. IL-1β and damage-associated molecules released during podocyte pyroptosis may further shape the local inflammatory milieu (58, 60) and may converge functionally with NLRP3-related responses in other kidney cells (66). Macrophages take up both EV receptor and donor roles across multiple independent studies, and this review therefore treats them as a candidate integration hub. Whether they occupy a central position awaits experimental verification. Macrophage EVs differ in cargo and effect according to activation state: EVs from macrophages in pro-inflammatory states generally carry injury-promoting cargo (40, 54, 55, 58, 65, 68), whereas EVs from reparative macrophages can carry protective cargo (61, 62). Because macrophage subsets and activation states vary during DKD progression (43), their EV composition and return signals may also change, shifting the relative strength of injury-promoting and protective signals across the network.

Macrophages in the DKD kidney are considerably more heterogeneous: their functional states depend on cellular origin, kidney compartment, and disease stage and can form continuous or mixed activation profiles (43). Integration of multiple inputs and the direction of the resulting EV signal should therefore be interpreted in relation to a specific macrophage population and its cargo. Within the network hypothesis, injury-promoting EVs perpetuate inflammation and tissue damage, whereas protective EVs limit injury by suppressing pyroptosis or restoring autophagy (61, 62). Their relative strength may help determine whether inflammation resolves or fibrosis progresses. TEC-derived EVs can also propagate an injury-associated phenotype within the TEC population (56), while EVs from CD4+ T cells induce TEC apoptosis (72), extending the network to adaptive immunity. A network containing both amplification and protective regulation offers one explanation for persistent inflammation that a linear cytokine cascade cannot fully capture. Even if the initiating metabolic disturbance subsides, established inflammatory and injury-promoting signals may persist or expand through multicellular communication, helping early injury progress to sustained DKD.

The integrated network hypothesis generates several experimentally testable predictions. If the LRG1/TRAIL axis is an important bidirectional injury pathway between TECs and macrophages, selective attenuation of EV-associated LRG1 signaling from TECs should reduce both macrophage activation and the TRAIL-related return signal. Determining whether that return signal alters subsequent TEC EV output would establish whether the chain becomes a self-sustaining feedback loop (40). If podocytes and macrophages communicate directly through EVs, cell-of-origin-specific vesicles should reach the predicted recipient *in vivo* and elicit a functional effect that is attenuated by source- or recipient-specific perturbation (57, 58). Finally, if macrophages integrate inputs from different resident kidney cells, their EV cargo and functional output should vary with the source of the input, macrophage state, and disease stage, and should track the relative dominance of injury-promoting or protective return signals (43, 58, 61, 62). Taken together, the preceding axes show three levels of relationship. LRG1/TRAIL has its forward and return actions connected within a single experimental system and represents experimentally validated communication. The podocyte–macrophage and mesangial cell–macrophage relationships each hold in both directions but have not been connected, and represent connections inferred across studies. This review accordingly proposes a network-level hypothesis in which these axes are linked through macrophage state and its signal output, jointly influencing the progression of inflammation and injury (**Figure 3**).

**Figure 3:**
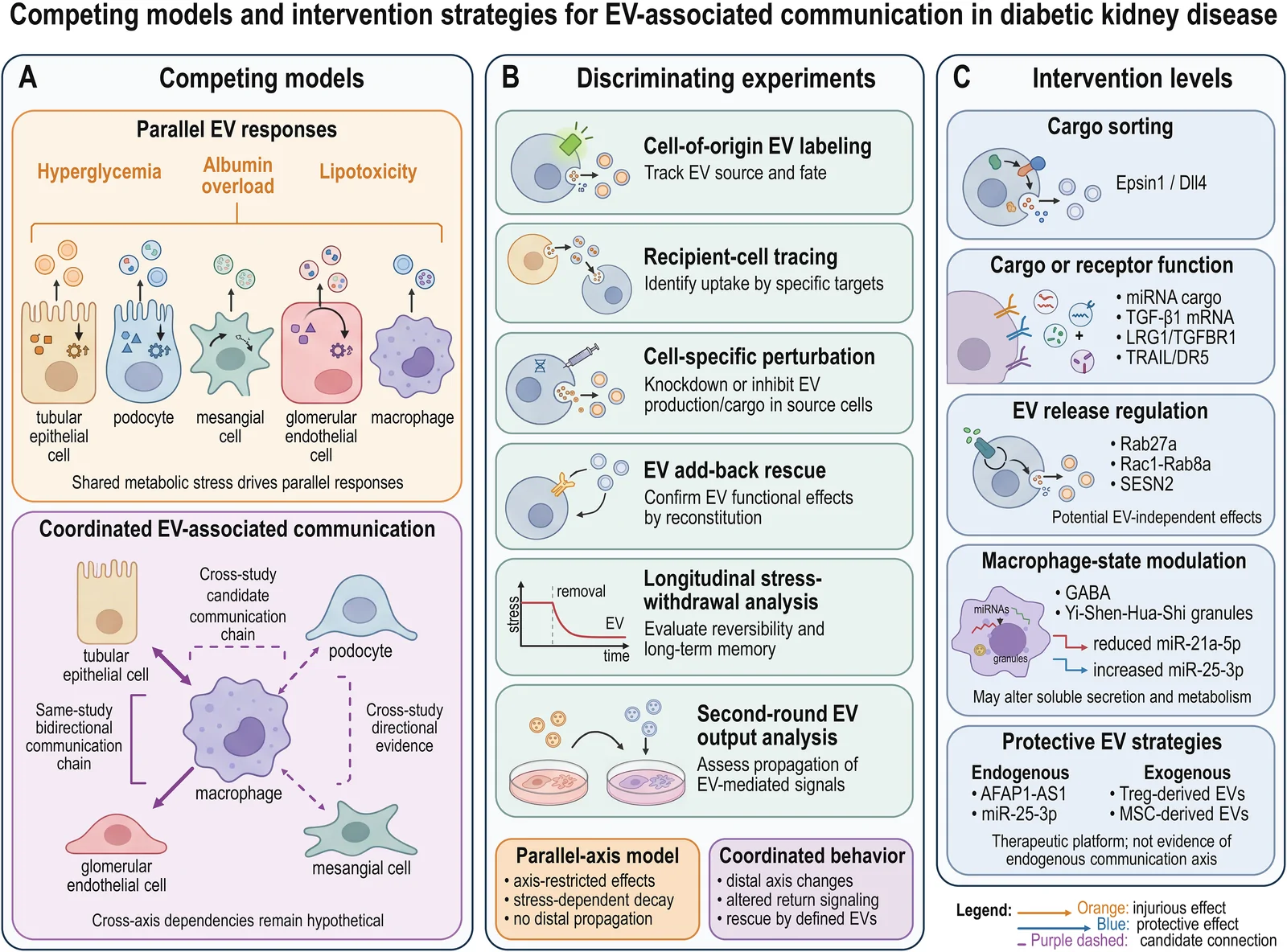
Competing models, discriminating experiments, and intervention levels for extracellular-vesicle-associated communication in diabetic kidney disease. **(A)** Independent EV responses to shared metabolic stressors are contrasted with coordinated EV-associated intercellular communication. Except for the same-study TEC–macrophage bidirectional chain, cross-axis coordination remains hypothetical. **(B)** Proposed discriminating experiments include cell-of-origin EV labeling, recipient-cell tracing, cell-specific perturbation, EV add-back rescue, longitudinal stress-withdrawal analysis, and second-round EV-output analysis. **(C)** Potential intervention levels include cargo sorting, cargo or receptor function, EV release, macrophage-state modulation, and endogenous or exogenous protective EV strategies. These approaches remain preclinical and may also exert EV-independent effects. DKD, diabetic kidney disease; EV, extracellular vesicle; MSC, mesenchymal stromal cell; TEC, tubular epithelial cell.

## 5 Potential interventions derived from the EV communication network hypothesis

The evidence reviewed above suggests that several EV-associated donor-recipient axes in DKD may be linked functionally through macrophages and shared effector modules such as NF-κB, NLRP3, and TGF-β/Smad. Their aggregate influence may also depend on the relative strength of injury-promoting and protective signals. Some of these approaches are presently more valuable as mechanistic tests than as therapies. Their translational potential can be assessed only after cell-type-specific efficacy *in vivo*, safety, and delivery feasibility have been established (**Table 2**; **Figure 3**).

**Table 2:** Potential strategies targeting EV-associated intercellular communication.

Intervention strategy	Representative target or agent	Primary intervention point	Principal experimental result	Evidence stage	Ref.
Cargo-sorting intervention	Epsin1/Dll4	Reduces Dll4 sorting into TEC-derived EVs	Attenuates macrophage Notch1 activation and pro-inflammatory responses	Comparatively strong mechanistic validation; acts mainly on the TEC-to-macrophage axis	(51)
Cargo-function intervention	miR-19b-3p, miR-199a-5p, miR-7002-5p, miR-21-5p, and TGF-β1 mRNA	Inhibits a defined EV cargo or its expression in donor cells	Reduces macrophage activation, impaired TEC autophagy, podocyte pyroptosis, and mesangial fibrosis	Preclinical mechanistic evidence; intervention level and cargo specificity differ among studies	(48, 49, 54, 58, 65)
Regulation of EV release	Rab27a, Rac1-Rab8a, and SESN2	Reduces TEC-derived EV output or directs multivesicular bodies toward the autophagy-lysosome pathway	Lowers inflammatory indices and attenuates kidney injury	Supportive preclinical evidence; targets also regulate membrane trafficking, autophagy, or stress responses independently of EVs	(73–75)
Modulation of macrophage state	GABA and Yi-Shen-Hua-Shi granules	Changes experimental macrophage state and EV cargo composition	Reduces injury-promoting miRNA; in the GABA model, also increases the miR-25-3p-related protective signal and attenuates podocyte injury	Preclinical intervention evidence; may also alter soluble secretion and cellular metabolism, and cross-axis effects remain untested	(60, 76)
Endogenous protective EV mechanism	EVs from reparative macrophages: lncRNA AFAP1-AS1 and miR-25-3p	Suppresses podocyte pyroptosis or improves autophagy	Improves high-glucose-associated podocyte injury phenotypes	State-related mechanistic model; supports a protective return signal but does not establish dynamic balance across the network	(61, 62)
Exogenous engineered EV formulation	RGD-Treg-Exos/miR-218-5p	Targets podocytes and promotes mitophagy	Attenuates podocyte injury and kidney damage in diabetic mice	Preclinical therapeutic-delivery evidence; does not establish an endogenous Treg-to-podocyte communication edge	(77)
Exogenous mesenchymal stromal cell (MSC)-EV formulation	MSC-derived EVs and multi-cargo signals including miR-99b-5p	Regulates autophagy, fibrosis-associated cell states, YAP, and ferroptosis	Attenuates inflammation, fibrosis, and kidney injury	Preclinical studies of diabetes-related kidney injury; multi-cargo and systemic effects limit direct intrarenal attribution, and no human intervention evidence exists	(78–82)

### 5.1 Restricting pathogenic cargo sorting

Blocking the entry of pathogenic cargo into EVs within the donor cell may preserve more physiological communication than broadly suppressing EV release. EPS15 interacting protein 1 (Epsin1) is currently the most thoroughly validated EV cargo-sorting target in DKD models. Under high-glucose conditions, Epsin1 sorts Dll4 into TEC-derived EVs. Epsin1 knockdown lowers EV Dll4, followed by reduced Notch homolog 1 (Notch1) activation and lower TNF-α, IL-6, and iNOS expression in macrophages (51). *In vivo*, Epsin1 knockdown also reduces macrophage infiltration and pro-inflammatory marker expression. Because this intervention decreases EV loading of Dll4 without changing total Dll4 expression in the donor cell, it has comparatively high pathway specificity. The sorting mechanisms for other pathogenic cargoes, including miR-19b-3p, miR-199a-5p, CCL2 mRNA, and LRG1, remain unknown.

### 5.2 Attenuating pathogenic cargo function

Interventions directed at a specific cargo can act by changing its expression in the donor cell, its loading into EVs, or its action in the recipient cell. Several forward and return signals in the proposed network depend on particular miRNA or mRNA cargoes. In the forward direction, miR-19b-3p from TEC-derived EVs enters macrophages and suppresses suppressor of cytokine signaling 1 (SOCS1), relieving inhibition of NF-κB and inducing pro-inflammatory macrophage markers and expression of IL-6, CCL2, TNF-α, and iNOS. Inhibition of miR-19b-3p reduces tubulointerstitial inflammation in a proteinuria model (48). In patients with DKD, urinary EV miR-19b-3p is inversely correlated with renal SOCS1 expression, providing evidence of clinical relevance for this axis. In the return direction, TGF-β1 knockdown in donor macrophages lowers TGF-β1 mRNA in their EVs and attenuates Smad3 activation and extracellular matrix deposition in mesangial cells (65), supporting a causal contribution of this cargo to profibrotic macrophage-to-mesangial-cell communication. Multiple EV cargoes act through different upstream mechanisms but converge on common inflammatory modules such as NF-κB, so parallel inputs and pathway redundancy may limit the overall effect of targeting a single cargo. Their primary value is to test the causal contribution of a defined cargo to a communication axis and to determine whether that cargo is a tractable intervention target.

### 5.3 Reducing EV release

EV biogenesis and release are central to inflammatory communication because a single vesicle population can carry several pathogenic signals. DKD studies indicate at multiple levels that altering TEC-derived EV release affects inflammation and kidney injury. Rab27a inhibition in TECs reduces EV secretion, retains relatively more miR-26a-5p within the donor cell, and attenuates CHAC1/NF-κB signaling (73). Proximal-tubule-specific deletion of Rac1 inhibits Rab8a-associated EV release and lowers both renal EV abundance and inflammatory indices (74). The Rac1 study used proximal-tubule-specific conditional knockout, one of the few cell-specific interventions among the studies reviewed here. SESN2 overexpression directs multivesicular bodies toward the autophagy-lysosome pathway and reduces TEC-derived EV release (75). Together, these findings support a role for TEC-derived EVs in inflammatory signaling in DKD and identify EV biogenesis and release as potential intervention points. However, Rab27a, Rac1, and SESN2 also participate in membrane trafficking, autophagy, and cellular stress responses. The protection observed in current studies cannot therefore be attributed entirely to reduced EV release. Systemic manipulation of these pathways could also disrupt physiological EV communication in other cells and organs, underscoring the need for cell- and tissue-selective approaches.

### 5.4 Modulating macrophage activation states

Changing macrophage state may have a broader influence because it can simultaneously alter several EV cargoes and their functional output, thereby shifting macrophage-derived return signals toward injury or protection. In podocyte-injury models, γ-aminobutyric acid (GABA) altered macrophage activation markers under high-glucose conditions, decreased EV miR-21a-5p, and increased EV miR-25-3p (76). The same intervention weakened the miR-21a-5p-mediated return signal that promotes podocyte pyroptosis and strengthened the miR-25-3p-mediated return signal that restores podocyte autophagy. Yi-Shen-Hua-Shi granules similarly reduced pro-inflammatory macrophage markers and EV miR-21a-5p in db/db mice and related cell models, accompanied by less podocyte injury (60). These findings indicate that changing macrophage activation can remodel several EV cargoes at once and redirect their effects. Macrophage activation *in vivo* is continuous, mixed, and stage-dependent, however, and some reparative states can contribute to fibrosis under specific conditions. Macrophage-directed interventions must therefore be evaluated in defined subsets and interpreted alongside EV cargo composition and renal pathology.

### 5.5 Enhancing protective EV signals

Enhancing protective EVs or their cargo offers a complementary way to alter network balance. Some protective signals act on the same or adjacent molecular nodes as injury-promoting pathways and may limit pathogenic amplification by suppressing inflammation, restoring autophagy, or reducing cell damage. Reparative macrophages provide two examples of protective EV cargo. Both cargoes act in the macrophage-to-podocyte direction and oppose the pro-pyroptotic effect of miR-21-5p from macrophages in a pro-inflammatory state. Thus, EVs produced by different macrophage states may exert countervailing effects on podocyte injury. Exogenous EVs can also serve as multi-cargo delivery platforms. Arginine-glycine-aspartate (RGD)-modified regulatory T cell (Treg)-derived engineered EV preparations deliver miR-218-5p to podocytes, restore mitophagy, and reduce kidney injury in diabetic mice (77). Mesenchymal stromal cell-derived EVs act on several injury pathways and have shown anti-inflammatory, antifibrotic, and cytoprotective effects in preclinical studies. Reported mechanisms include restoration of autophagy, modulation of fibrosis-associated macrophage populations, promotion of Yes-associated protein (YAP) degradation, and suppression of ferroptosis (78–81). Increasing protective EV signals may instead rebalance the network by limiting pyroptosis, improving autophagy, and preserving cellular homeostasis. These approaches represent distinct therapeutic rationales rather than a direct comparison of efficacy, and both face substantial barriers between preclinical models and clinical use (82).

## 6 Discussion: evidence boundaries, translational implications, and unresolved questions

Integrating dispersed EV-mediated communication axes into a cytokine network offers a multicellular perspective on persistent inflammation and progressive injury in DKD. Current evidence, however, still consists largely of separate communication axes; their spatial relationships, temporal behavior, and causal dependence remain uncertain. Methodologically, many studies isolate EVs by differential centrifugation or precipitation kits, which do not completely separate soluble cytokines from EV-associated material (32). Some effects attributed to EVs may therefore arise from co-isolated free mediators. Applying these criteria separates axes that are supported by source-defined vesicles acting on a specified recipient from those, such as the monocyte microvesicle axis (64), in which the vesicle-associated signal remains to be distinguished from co-isolated material. Future studies should use more stringent isolation and control strategies, combine cargo localization with functional perturbation, and exclude co-isolated proteins, nucleic acids, and soluble factors as far as possible (32). EV targeting and delivery efficiency may also be altered by a surface protein corona or by endosomal retention after uptake. For miRNA- or lncRNA-mediated mechanisms, measuring total EV RNA and recipient-cell uptake is insufficient. The RNA must be shown to associate with EVs, reach the recipient-cell cytosol, and produce the proposed functional effect.

Direct evidence for a closed loop *in vivo* is generally absent. The LRG1/TRAIL study included a DKD animal model, primary cells, and cell lines, but receptor-dependent testing and intervention in both directions were performed mainly in cellular systems. Cell-type-specific interruption of bidirectional communication has not been demonstrated in an animal model of DKD (40). The forward and return signals proposed between podocytes and macrophages came from different laboratories and different models (57, 58) and have never been linked into a closed loop within one *in vivo* experiment. No *in vivo* tracing study has shown podocyte-specific EVs crossing the glomerular filtration barrier to reach macrophages. The route by which this communication could occur therefore remains unresolved.

Most studies sample one or a few time points. Direct dynamic evidence is lacking for how the relative contribution of injury-promoting and protective signals changes across disease stages (58, 60–62) or whether the associated communication persists after the initiating metabolic stress has diminished. Macrophage populations change during DKD progression (43), but systematic studies have not established whether EV cargo changes in parallel with macrophage state and disease stage. The evolving balance between injurious and protective signals and its contribution to disease progression must therefore be tested longitudinally. A binary classification of macrophage activation cannot capture macrophage heterogeneity *in vivo* (43), and no experimental data show how macrophages integrate multiple parallel inputs (48–50, 57) to determine EV cargo output. These fundamental gaps also constrain therapeutic translation.

Multiple forward pathways converge on shared nodes such as NF-κB (48–50, 57). Blocking one cargo (48, 49, 54, 58) or the Epsin1/Dll4 sorting pathway (51) may therefore affect the corresponding axis while leaving parallel injury-promoting signals intact. Conversely, targets used to inhibit EV release systemically (73–75) or alter macrophage polarization (60, 76) are broadly expressed and lack kidney or cell-type specificity. Validating this network depends on three types of experiment in particular. Cell-of-origin tracing establishes the donor cell identity and the reach of the vesicles *in vivo*. Cell-specific perturbation replaces systemic intervention with conditional knockout in a defined cell population. EV add-back restores vesicles of defined origin in the knockout background and tests whether the phenotype is recovered. Single-cell and spatial transcriptomics and single-vesicle analysis complement these experiments by resolving how the relevant cell populations and vesicle subsets change over the course of disease (83, 84).

Urinary EVs are attractive as a non-invasive window on intrarenal communication, and their molecular changes correlate with DKD severity (41, 42, 85). Their composition reflects their cells of origin, which in urine are mainly the tubular epithelium and the urothelium, whereas glomerular vesicles must cross the filtration barrier and may be taken up or degraded within the tubular lumen. Urinary EV changes therefore weight the tubular compartment more heavily than the glomerular one. One study did correlate urinary small-EV C-C motif chemokine ligand 21 (CCL21) mRNA with the number of CD3-positive sites in kidney tissue from the same patients, but the analysis was cross-sectional, the histological comparison was restricted to small subgroups, and the control kidney tissue came from patients with renal cancer (86). Demonstrating that urinary EVs report a defined intrarenal communication axis requires longitudinal sampling of urine and kidney tissue from the same patients, cell-of-origin-specific markers to assign each vesicle population, and analysis of whether urinary EV changes track the activity of that axis (**Figure 3**).

## 7 Conclusions

Existing studies support several EV-associated donor–recipient communication axes in DKD. The LRG1/TRAIL chain between tubular epithelial cells and macrophages has been validated in both directions within a single experimental system, whereas the podocyte and mesangial cell axes each hold in both directions without having been connected. These axes converge on shared effector modules including NF-κB, NLRP3, and TGF-β/Smad, and are driven by hyperglycaemia, albumin overload, and lipotoxicity. This review accordingly proposes that the axes are linked through macrophage state and its signal output, with injury-amplifying and protective signals acting in parallel to shape the progression of inflammation and fibrosis. Testing this hypothesis requires cell-of-origin tracing, cell-specific perturbation, and EV add-back experiments.

## Glossary

A20: tumor necrosis factor alpha-induced protein 3
AFAP1-AS1: actin filament-associated protein 1 antisense RNA 1
APOC1: apolipoprotein C1
AQP2: aquaporin 2
Atg9b: autophagy-related protein 9B
CCL2: C-C motif chemokine ligand 2
CCL21: C-C motif chemokine ligand 21
CD4: cluster of differentiation 4
CHAC1: ChaC glutathione-specific gamma-glutamylcyclotransferase 1
circRNA: circular RNA
CXCL7: C-X-C motif chemokine ligand 7
CXCL12: C-X-C motif chemokine ligand 12
DKD: diabetic kidney disease
Dll4: Delta-like ligand 4
DR5: death receptor 5
DUSP1: dual-specificity phosphatase 1
Epsin1: EPS15-interacting protein 1
EV: extracellular vesicle
EZH2: enhancer of zeste homolog 2
GABA: γ-aminobutyric acid
GEC: glomerular endothelial cell
HIF-1α: hypoxia-inducible factor 1α
IL: interleukin
iNOS: inducible nitric oxide synthase
lncRNA: long noncoding RNA
LRG1: leucine-rich alpha-2-glycoprotein 1
m6A: N6-methyladenosine
M1: classically activated macrophage state as defined experimentally
M2: alternatively activated macrophage state as defined experimentally
MCP-1: monocyte chemoattractant protein 1
METTL14: methyltransferase-like 14
MISEV: Minimal Information for Studies of Extracellular Vesicles
miRNA: microRNA
mRNA: messenger RNA
MSC: mesenchymal stromal cell
NF-κB: nuclear factor κB
NLRP3: NLR family pyrin domain-containing 3
Notch1: neurogenic locus notch homolog protein 1
NOX4: NADPH oxidase 4
PAQR3: progestin and adipoQ receptor family member 3
PRKAR2B: protein kinase cAMP-dependent type II regulatory subunit beta
RAAS: renin-angiotensin-aldosterone system
Rab27a: Ras-related protein Rab-27A
Rab8a: Ras-related protein Rab-8A
Rac1: Ras-related C3 botulinum toxin substrate 1
RGD: arginine-glycine-aspartate
ROS: reactive oxygen species
SESN2: sestrin 2
SMAD3: SMAD family member 3
SOCS1: suppressor of cytokine signaling 1
TEC: tubular epithelial cell
TGF-β1: transforming growth factor-β1
TGFβR1: transforming growth factor-β receptor 1
THP-1: human monocytic leukemia cell line THP-1
TLR4: Toll-like receptor 4
TNF-α: tumor necrosis factor-α
TRAIL: TNF-related apoptosis-inducing ligand
Treg: regulatory T cell
VEGF: vascular endothelial growth factor
YAP: Yes-associated protein
