# Supplementary material for: Extracellular vesicle–cytokine communication in diabetic kidney disease: an integrated network linking renal resident cells and macrophages
Source: Front Immunol. 2026 Oct 8;17:1967300. doi: 10.3389/fimmu.2026.1967300 (PMC13688021; doi:10.3389/fimmu.2026.1967300)
Supplement: Supplementary file 1 [file Table1.docx]

Supplementary Table 1. EV methodological quality of the studies cited in this review. Each row is one study, cited by reference number.

| **Ref.** | **Study design** | **EV separation** | **EV characterization** | **Vesicle-depleted**  **control** | **Cargo localization**  **/ protection** | **Uptake or**  **transfer** | **Cargo-specific**  **perturbation** | Overall EV attribution |
| --- | --- | --- | --- | --- | --- | --- | --- | --- |
| 6 | Not an EV study | NA | NA | NA | NA | NA | NA | NA |
| 8 | Not an EV study | NA | NA | NA | NA | NA | NA | NA |
| 19 | In vitro, ex vivo, human fluids | ExoQuick kit | NTA (one category) | No / culture condition only | Yes | Partial | Donor only | A2 |
| 36 | In vitro | differential centrifugation + UC | No | No | NR | NR | NR | A3 |
| 37 | In vitro | differential centrifugation + UC | WB: CD63, CD9 + calnexin−; TEM | No / culture condition only | NR | Yes | Donor only | A2 |
| 38 | In vitro | differential centrifugation + UC | WB: CD63, CD81; TEM | No / culture condition only | NR | Yes | Receptor only | A2 |
| 39 | In vitro + in vivo | precipitation kit | WB: CD63; TEM | Yes | NR | Yes | Donor only (no rescue) | A1 |
| 40 (forward arm) | In vitro + in vivo | differential UC (cell-line EVs); ExoQuick-TC (primary-cell EVs), as reported | WB: CD63 + TSG101, ARF6 + calnexin−; TEM; NTA | Yes (boiled EVs) | Partial (immunogold, LRG1) | Yes | Receptor only, forward arm only | A1 |
| 40 (return arm) | In vitro | differential UC (macrophage-derived EVm, as reported); ExoQuick-TC (primary-cell EVs) | WB: CD63 + TSG101, ARF6 + calnexin−; TEM; NTA | No | Partial (immunogold, TRAIL) | Yes | NR | A2 |
| 41 | Clinical | UC | WB: CD9 + podocalyxin; TEM; NTA | No | NR | NR | NR | A2 |
| 42 | Clinical | UC; precipitation kit | Partial (cross-reference only) | No | NR | NR | NR | A2 |
| 48 | In vitro + in vivo | differential UC | WB: CD9, CD63 + ALIX | No / culture condition only | NR | Yes | Donor only | A2 |
| 49 | In vitro + in vivo | precipitation kit (ExoQuick) | TEM; NTA | Yes | NR | Yes | Donor only (no rescue) | A1 |
| 50 | In vitro + in vivo | differential UC + density gradient | WB: CD9, CD63 + ALIX; NTA | No / culture condition only | NR | Yes | Donor only | A2 |
| 51 | In vitro + in vivo | differential centrifugation + filtration + UC | WB: CD9, CD81 + ALIX + calnexin− | No / culture condition only | NR | Yes | Donor only | A2 |
| 52 | In vitro + in vivo | differential UC + density gradient | WB: CD63 + TSG101 + Grp94− | No | NR | Yes | Receptor only | A2 |
| 53 | In vitro + in vivo | differential UC + density gradient | TEM | No / culture condition only | NR | Yes | Donor only | A2 |
| 54 | In vitro + in vivo | precipitation kit; density gradient; immunoaffinity | WB: CD63 + ALIX, TSG101; TEM; NTA | Yes | NR | Yes | Donor only (no rescue) | A1 |
| 55 | In vitro | PEG precipitation + immunoaffinity | WB: CD9, CD81 + histone H3− | No / culture condition only | NR | Yes | NR | A2 |
| 56 | In vitro + in vivo | UC + immunoaffinity | nano-flow cytometry | No / culture condition only | NR | Yes | Receptor only | A2 |
| 57 | In vitro | differential centrifugation | WB: CD63 + TSG101 + calnexin− | No | NR | Yes | Donor only | A2 |
| 58 | In vitro + in vivo | UC + density gradient | EM | No | NR | Partial | Donor only | A2 |
| 60 | In vitro + in vivo | precipitation kit (Wako) | WB: CD9, CD63, CD81; TEM | No / culture condition only | NR | NR | NR | A2 |
| 61 | In vitro + clinical | UC + 0.22 um filtration | WB: CD9, CD63, CD81 + calnexin−; TEM; NTA | Yes | NR | Partial | Donor only | A2 |
| 62 | In vitro | UC | WB: CD9, CD63; TEM | Yes | NR | Yes | Donor only (no rescue) | A1 |
| 63 | In vitro + in vivo | precipitation kit + PS-affinity | NTA (one category) | No / culture condition only | NR | Yes | NR | A2 |
| 64 | In vitro | differential centrifugation + UC | No | No | NR | NR | NR | A3 |
| 65 | In vitro + in vivo | precipitation kit | WB: CD63 + TSG101 + calnexin−; TEM | No | NR | Yes | Donor only | A2 |
| 66 | In vitro + in vivo | differential UC | WB: CD9, CD63 + TSG101; TEM | No / culture condition only | NR | Yes | NR | A2 |
| 67 | Clinical + in vitro | precipitation kit (ExoQuick) | Partial (cross-reference only) | No / culture condition only | NR | NR | NR | A2 |
| 68 | In vitro | UC | WB: CD63, CD81 + TSG101; TEM; NTA | Yes | NR | Yes | Donor only (no rescue) | A1 |
| 69 | In vitro + in vivo | precipitation kit (ExoQuick) | WB: CD9 + flotillin-1 + calnexin−; TEM | No / culture condition only | NR | Yes | Donor only | A2 |
| 70 | Clinical + in vitro | differential UC; precipitation kit | WB: CD9, CD63; TEM | No | NR | Partial | Donor only | A2 |
| 71 | In vitro + in vivo | differential centrifugation; flow cytometry | WB: CD61, CD63; EM | No / culture condition only | NR | Yes | Receptor only | A2 |
| 72 | In vitro + in vivo | UC | WB: CD63, CD81 + TSG101; TEM; nano-flow cytometry | No | NR | NR | NR | A2 |
| 73 | In vitro + in vivo (animal part descriptive) | precipitation kit (ExoQuick-TC) | No | Yes | NR | NR | Donor only | A3 |
| 74 | In vitro + in vivo | UC | TEM; NTA | Yes | NR | Yes | Donor only (no rescue) | A1 |
| 75 | In vitro + in vivo | UC; precipitation kit | TEM; NTA | Yes | NR | Yes | Donor only (no rescue) | A1 |
| 76 | In vitro | NR | WB: CD9, CD63; TEM | Yes | NR | Yes | Donor only (no rescue) | A1 |
| 77 | In vitro + in vivo | UC | WB: CD63 + ALIX, TSG101 + calnexin− | Yes | NR | Yes | Donor only (no rescue) | A1 |
| 78 | In vivo | differential UC + density gradient | WB: CD63, CD81; TEM | No / culture condition only | NR | Partial | Receptor only | A2 |
| 79 | In vivo + in vitro | differential centrifugation + UC | WB: CD9, CD63, CD81 + calnexin−, cytochrome C− | No | Partial | Yes | Donor only | A2 |
| 80 | In vitro + in vivo | differential UC + density gradient | WB: CD9 + TSG101, ALIX + calnexin−; DLS | No / culture condition only | NR | Yes | Donor only | A2 |
| 81 | In vivo + in vitro | differential UC + density gradient | WB: CD9, CD63 + TSG101; NTA | No | NR | Partial | Receptor only | A2 |
| 85 | Clinical | differential UC + density gradient | WB: CD63, CD9 + podocalyxin; TEM; IEM; NTA | No | NR | NR | NR | A2 |
| 86 | Clinical + in vivo | differential UC + density gradient | WB: CD63, CD81 + ALIX; TEM | No | Yes | NR | NR | A2 |

Abbreviations and grading. NA, not applicable; NR, not reported. UC, ultracentrifugation. No / culture condition only: EV-depleted serum or medium was used as a culture condition, which is not a control for an EV-dependent effect. EV characterization follows MISEV2023 (Welsh et al., J Extracell Vesicles 2024): Yes, two or more of the five MISEV2023 protein categories, particle size or concentration, or single-vesicle morphology covered; Partial, one; No, none. Cargo localization / protection: whether the cargo is enclosed in the vesicle (protease or RNase protection with detergent lysis, or non-permeabilized flow cytometry); immunogold alone is Partial and does not count as characterization, because it does not separate surface-bound from luminal cargo. Overall EV attribution: A1, characterization plus a valid vesicle-depleted control plus demonstrated uptake or transfer; A2, some of these; A3, not supported. Where a study reports conclusions beyond its assays, the assays are recorded; ref 40 is A2 for its return arm because DR5 was assessed by expression, not by receptor blockade. Cargo-specific perturbation is graded separately: Donor only, donor-side loss-of-function without rescue; Receptor only, recipient-side blockade alone; Both, re-expression in the same knockdown background; NR, none reported.
